# Exploring the Association Between Genetic Polymorphisms in Genes Involved in Craniofacial Development and Isolated Tooth Agenesis

**DOI:** 10.3389/fphys.2021.723105

**Published:** 2021-09-01

**Authors:** Erika Calvano Küchler, Caio Luiz Bitencourt Reis, Alice Corrêa Silva-Sousa, Guido Artemio Marañón-Vásquez, Mirian Aiko Nakane Matsumoto, Aline Sebastiani, Rafaela Scariot, Eva Paddenberg, Peter Proff, Christian Kirschneck

**Affiliations:** ^1^Department of Orthodontics, University Medical Centre of Regensburg, Regensburg, Germany; ^2^Department of Pediatric Dentistry, School of Dentistry of Ribeirão Preto, University of São Paulo, Ribeirão Preto, Brazil; ^3^Department of Restorative Dentistry, School of Dentistry of Ribeirão Preto, University of São Paulo, Ribeirão Preto, Brazil; ^4^Department of Pediatric Dentistry and Orthodontics, School of Dentistry, Federal University of Rio de Janeiro, Rio de Janeiro, Brazil; ^5^Department of Stomatology, Federal University of Paraná, Curitiba, Brazil

**Keywords:** genetic polymorphisms, craniofacial development, dental anomaly, tooth agenesis, single nucelotide polymorphisms

## Abstract

Tooth agenesis is a common congenital anomaly in humans and is more common in oral cleft patients than in the general population. Many previous studies suggested that oral cleft and tooth agenesis share a similar genetic background. Therefore, this study explored the association between isolated tooth agenesis and genetic polymorphisms in genes that are crucial for craniofacial and tooth development. Panoramic radiographs, anamnesis, and genomic DNA from 273 patients were included. Patients were classified as tooth agenesis present, when at least one permanent tooth was congenitally missing. Patients with syndromes and oral cleft were excluded. Only unrelated patients were included. The genetic polymorphisms in *BMP2* (rs235768 and rs1005464), *BMP4* (rs17563), *RUNX2* (rs59983488 and rs1200425), and *SMAD6* (rs3934908 and rs2119261) were genotyped by real-time polymerase chain reaction. Genotype and allele distributions were compared between the tooth agenesis phenotypes and controls by Chi-square test. Haplotype and diplotype analysis were also performed, in addition to multivariate analysis (alpha of 0.05). A total of 86 tooth agenesis cases and 187 controls were evaluated. For the rs235768 in *BMP2*, patients carrying TT genotype have higher chance to present tooth agenesis [*p* < 0.001; prevalence ratio (PR) = 8.29; 95% confidence interval (CI) = 4.26–16.10]. The TT genotype in rs3934908 (*SMAD6*) was associated with higher chance to present third molar agenesis (*p* = 0.023; PR = 3.25; 95% CI = 1.17–8.99). *BMP2* was also associated in haplotype and diplotype analysis with tooth agenesis. In conclusion, genetic polymorphisms in *BMP2* and *SMAD6* were associated with isolated tooth agenesis.

## Introduction

Isolated tooth agenesis (or congenitally missing teeth) is one of the most common congenital defects in humans, which affects approximately 20% of the average worldwide population (Vastardis, 2000). Tooth agenesis can be classified into two main types: non-syndromic and syndromic. Non-syndromic tooth agenesis involves a congenitally missing permanent or primary tooth or teeth in an isolated form without any other major birth defects, such as oral cleft and syndromes. Isolated tooth agenesis occurs in both aches (maxilla and mandible) and can affect any type of teeth, although the most commonly affected teeth are third molars, maxillary lateral incisors, and premolar (Küchler et al., 2008a,b). Syndromic tooth agenesis refers to congenitally missing teeth associated with syndromes and oral clefts (such as cleft lip, clef palate, and cleft lip with palate) (Lu et al., 2016). Tooth agenesis is often observed in individuals with oral clefts (in the cleft area and also in non-cleft areas) and their non-affected family members (Küchler et al., 2011). Several observational epidemiological and genetic studies suggest that oral clefts and isolated tooth agenesis share a similar genetic background (Phan et al., 2016).

The bone morphogenetic protein (BMP) family, comprising an extensive group of phylogenetically conserved growth factors, such as BMP2 and BMP4, which plays an important role during tooth development (Zhang et al., 2005; Saadi et al., 2013; Taşlı et al., 2014; Yuan et al., 2015). Interestingly, genetic polymorphisms in *BMP2* and *BMP4* have been associated with both, isolated tooth agenesis (Antunes et al., 2012; Gong et al., 2015) and oral clefts (Antunes et al., 2013; Saket et al., 2016; Bahrami et al., 2020). Mothers against decapentaplegic homolog 6 (SMAD6) belongs to the SMAD family, which are important signaling pathway proteins during craniofacial development (Estrada et al., 2011; Suzuki et al., 2020). SMAD6 is known to inhibits BMP signaling in the nucleus by interacting with transcription repressors (Wu et al., 2016). Another molecule with a crucial role in craniofacial development is Runt-related transcription factor (RUNX2). This gene has been identified as essential for tooth formation (Camilleri and McDonald, 2006). Therefore, this study explored the association between isolated permanent tooth agenesis and genetic polymorphisms in genes that are crucial for tooth and craniofacial development.

## Materials and Methods

### Sample

The study protocol was reviewed and approved by the local Ethics Committee (no. 01451418.3.0000.5419). Informed consent was obtained from all participating individuals or parents/legal guardians during the dental appointment and the assent was also obtained from children. The guideline Strengthening the Reporting of Genetic Association (STREGA) was followed for this study (Little et al., 2009).

Pre-dental treatment records including anamnesis and panoramic radiographs from patients undergoing dental treatment in universities and private clinics in Curitiba, Paraná state and Ribeirão Preto, São Paulo state (both cities located in Brazil) were evaluated. The sample consisted of biologically unrelated individuals aged 8–43 years old. The exclusion criteria included patients younger than 7 years of age, patients with syndromes, oral clefts, history of facial trauma or facial surgery, and records with missing radiographs in the tooth agenesis and control group.

### Determination of Tooth Agenesis Phenotype

The control and tooth agenesis cases were identified by the assessment of panoramic radiographs and treatment records by two trained dentists. All panoramic radiographs were examined using the same protocol and in all cases tooth agenesis was clearly evident from the panoramic radiographs alone (Küchler et al., 2008a,b). The inclusion criterion in the tooth agenesis group was that at least one permanent tooth was affected. Tooth agenesis was defined based on the age of subjects and when initial tooth formation should be visible in the radiographs (Küchler et al., 2008a,b). All controls had all permanent teeth, including third molars. Patients with tooth extraction were excluded.

Tooth agenesis cases were also divided into third molar agenesis and other permanent tooth agenesis subgroups for the analysis.

### Selection of Genetic Polymorphisms, DNA Extraction, and Genotyping

The selection of the genes was initially based on the screening of previously published studies suggesting that these genes are involved in the maxilla, mandible, and tooth development phenotypes. Then, potentially functional genetic polymorphisms were screened from the dbSNP database^1^ and SNPinfo,^2^ based on the following criteria: minor allele frequency (MAF) ≥10% in the global population, and classification of the genetic polymorphisms as potentially functional (for altering amino acid sequence of the protein product, or for occurring in the proximal promoter of the gene and potentially influencing gene expression, or previously associated with craniofacial phenotypes). The characteristics of the selected genetic polymorphisms are presented in Table 1.

**TABLE 1:** Characteristics of the selected genetic polymorphisms and previously reported associations with oral phenotypes.

Gene	Chromosome	Genetic polymorphism	Base change	Function	MAF	References
Bone morphogenetic protein 2 (*BMP2*)	20p12.3	rs1005464	A/G	Intron	0.194	Previously associated with dental crowding (Ting et al., 2011) and mandibular retrognathism (Küchler et al., 2021).
		rs235768	A/T	Missense (Arg > Ser)	0.676	Previously associated with mandibular retrognathism (Küchler et al., 2021)
Bone morphogenetic protein 4 (*BMP4*)	14q22.2	rs17563	A/G	Missense (Val > Ala)	0.454	Previously associated with isolated tooth agenesis (Antunes et al., 2012; Gong et al., 2015). May be a risk factor for oral clefts in Brazilians (Antunes et al., 2013; Bahrami et al., 2020)
Runt-related transcription factor 2 (*RUNX2*)	6p21.1	rs59983488	G/T	Upstream	0.179	This polymorphism was associated with maxillary protrusion (Küchler et al., 2021)
		rs1200425	A/G	Intron	0.448	Previously associated with skeletal malocclusion phenotypes (Küchler et al., 2021)
SMAD family member 6 (*SMAD6*)	15q22.31	rs2119261	C/T	Intron	0.419	Associated with the shape of the palatine rugae pattern (Silva-Sousa, 2021)
		rs3934908	C/T	Intron	0.436	Borderline association with the palatine rugae length asymmetry (Silva-Sousa, 2021).

*Obtained from databases: http://www.thermofisher.com and http://www.ncbi.nlm.nih.gov.*

*Arg, arginine; Ser, serine; Val, valine; Ala, alanine.*

For the genotyping analysis genomic DNA was used. The DNA was isolated from buccal epithelial cells by a rinse of saline solution. Briefly, the tubes with saliva were centrifuged and supernatant was discarded. Extraction solution (Tris–HCl 10 mmol/L, pH 7.8; EDTA 5 mmol/L; SDS 0.5%, 1 mL) and proteinase K (100 ng/mL) were added to the tube. Ammonium acetate also was added to remove non-digested proteins and the solution centrifuged. DNA was precipitated with isopropanol and washed with ethanol. DNA was then resuspended and quantified by spectrophotometry (NanoDrop 1000; Thermo Scientific, Wilmington, DE, United States) (Küchler et al., 2012).

The selected genetic polymorphisms were blindly genotyped *via* real-time polymerase chain reaction (PCR) StepOne^TM^ using TaqMan^TM^ technology (Applied Biosystems). The TaqMan technology uses extremely sensitive allele-specific probes (VIC^TM^ and FAM^TM^ dyes were used for the alleles). A negative control template was included in every reaction plate. In addition, 10% of samples were randomly selected for repeated analysis and the results showed 100% concordance. DNA samples that failed to be genotyped were excluded from further analyses. The success rates were as follows: rs235768 (*BMP2*) = 86.0%; rs1005464 (*BMP2*) = 84.9%; rs17563 (*BMP4*) = 84.2%; rs59983488 (*RUNX2*) = 85.5%; rs1200425 (*RUNX2*) = 83.8%; rs3934908 (*SMAD6*) = 84.9%; rs2119261 (*SMAD6*) = 84.2%.

### Statistical Analysis

Hardy–Weinberg Equilibrium (HWE) was assessed for each genetic polymorphism by Chi-square test.^3^ Chi-square test was also used to compare the allele and genotype distribution according to tooth agenesis phenotypes. Prevalence ratio (PR) and 95% confidence intervals (CI) were obtained. PLINK^4^ was used to compare haplotype frequencies between groups using Fisher’s exact test.

Multivariate Poisson regression adjusted by gender and ethnicity was done to evaluate genotypes in the co-dominant model and also diplotypes. Diplotype is a combination of two haplotypes that may be evaluated by an interaction term (Gatlin et al., 2009; Guo et al., 2017). Poisson regression was performed using SPSS Statistics Version 25.0 (IBM Corp., New York, NY, United States).

The significance level was determined as *p* < 0.05.

## Results

A total of 273 individuals (116 males and 157 females) was included. Eighty-six were included in the tooth agenesis group and 187 in the control group. In the tooth agenesis group, 53 individuals presented third molar agenesis (61.6%) and 42 (48.4%) presented other types of missing teeth. Gender and ethnicity were not associated with tooth agenesis (*p* > 0.05) (Supplementary Table 1).

Table 2 shows the allele distribution according to the groups. The allele T of the rs235768 in *BMP2* was associated with higher chance to present tooth agenesis in comparison with control group (*p* < 0.001; PR = 3.45; 95% CI = 2.54–4.70). In the subgroup analysis, the T allele was also associated with higher chance to present third molar agenesis (*p* < 0.001; PR = 4.30; 95% CI = 2.83–6.52) and other agenesis (*p* < 0.001; PR = 4.48; 95% CI = 2.78–7.21). The allele C of the rs3934908 in *SMAD6* was associated only with higher chance to present third molar agenesis (*p* = 0.036; PR = 1.43; CI 95% = 1.00–2.09).

**TABLE 2:** Allelic distribution between groups.

Gene	Genetic polymor phism	Allele	Control	Tooth agenesis	*p*	PR (95% CI)	Third molar agenesis	*p*	PR (95% CI)	Other agenesis	*p*	PR (95% CI)
*BMP2*	rs235768	A	233	42	**<0.001**	3.45 (2.54–4.70)	25	**<0.001**	4.30 (2.83–6.52)	20	**<0.001**	4.48 (2.78–7.21)
		T	91	104			65			50		
	rs1005464	G	249	112	>0.999	1.01 (0.72–1.42)	67	0.457	1.05 (0.68–1.62)	55	0.480	0.94 (0.56–1.58)
		A	73	30			21			15		
*BMP4*	rs17563	A	184	75	0.182	1.15 (0.87–1.51)	50	0.477	1.03 (0.71–1.50)	38	0.338	1.12 (0.73–1.72)
		G	134	67			38			32		
*RUNX2*	rs59983488	G	260	116	0.326	0.89 (0.60–1.31	73	0.467	0.93 (0.57–1.53)	56	0.344	0.82 (0.44–1.54)
		T	58	22			15			10		
	rs1200425	G	183	90	0.105	0.81 (0.61–1.09)	54	0.226	0.84 (0.56–1.24)	46	0.079	0.69 (0.43–1.11)
		A	135	50			32			22		
*SMAD6*	rs3934908	C	175	71	0.165	1.16 (0.88–1.52)	38	**0.036**	1.43 (1.00–2.09)	38	0.434	1.06 (0.70–1.61)
		T	145	73			50			34		
	rs2119261	C	179	81	0.390	0.94 (0.71–1.25)	51	0.415	0.93 (0.64–1.36)	40	0.383	0.90 (0.58–1.40)
		T	141	59			37			28		

*Fisher’s exact tests were performed. All comparisons were performed with control group as reference. Bold forms means statistical difference.*

*PR, prevalence ratio.*

Table 3 presents the haplotype frequency comparisons between groups. All haplotypes formed by the rs235768 and rs1005464 polymorphisms in *BMP2* were associated with tooth agenesis and tooth agenesis subgroups (*p* < 0.001). The haplotypes T–G and T–A were more frequent in tooth agenesis cases, while the haplotypes A–G and A–A were more frequent in controls.

**TABLE 3:** Haplotypes frequency comparisons between groups.

Genes	Genetic polymorphism	Haplotypes	Control	Tooth agenesis	*p*	Control	Third molar agenesis	*p*	Control	Other agenesis	*p*
*BMP2*	rs235768 and rs1005464	A–G	52.2	26.5	**<0.001**	51.9	24.0	**<0.001**	51.4	25.6	**<0.001**
		A–A	19.5	1.5	**<0.001**	19.8	2.0	**<0.001**	20.2	2.9	**<0.001**
		T–G	25.1	52.2	**<0.001**	25.4	52.0	**<0.001**	25.8	52.9	**<0.001**
		T–A	3.1	19.5	**<0.001**	2.8	21.7	**<0.001**	2.3	2.9	**<0.001**
*RUNX2*	rs59983488 and rs1200425	G–G	49.4	53.3	0.444	49.6	51.0	0.828	50.0	57.7	0.259
		G–A	32.4	30.4	0.683	32.1	31.5	0.914	31.8	26.5	0.411
		T–G	8.2	11.3	0.290	7.9	11.7	0.266	7.5	10.9	0.369
		T–A	9.9	4.8	0.072	10.1	5.6	0.197	10.5	4.6	0.143
*SMAD6*	rs3934908 and rs2119261	C–C	34.4	33.2	0.825	34.2	30.5	0.515	33.9	34.2	0.965
		C–T	20.4	15.3	0.197	20.4	12.6	0.094	20.8	17.2	0.508
		T–C	21.6	24.5	0.493	21.7	27.3	0.266	22.0	24.6	0.649
		T–T	23.5	26.8	0.454	23.5	29.4	0.254	23.1	23.9	0.896

*PLINK compares the frequencies between expected number of haplotypes by Fisher’s exact test. Bold forms means statistical difference.*

Table 4 shows the genotype distribution among groups in the co-dominant model. The rs235768 in *BMP2* was associated with tooth agenesis in univariate and multivariate analysis (*p* < 0.001). The genotype TT in rs3934908 in *SMAD6* was associated with an increased chance to present third molar agenesis (*p* = 0.023; PR = 3.25; CI 95% = 1.17–8.99).

**TABLE 4:** Genotypic distribution between groups.

Geno	Control	Tooth agenesis	p^u^	p^m^	PR^m^ (95% CI)	Third molar agenesis	p^u^	p^m^	PR^m^ (95% CI)	Other agenesis	p^u^	p^m^	PR^m^ (95% CI)
rs235768 (*BMP2*)													
AA	78 (48.1)	8 (11.0)	Ref.			4 (8.9)	Ref.			4 (11.1)	Ref.		
AT	77 (47.5)	26 (35.6)	**0.045**	**0.007**	2.76 (1.32–5.79)	17 (37.8)	**0.009**	**0.011**	3.89 (1.36–11.14)	12 (36.3)	0.067	0.068	2.77 (0.92–8.29)
TT	7 (4.3)	39 (53.4)	**<0.001**	**<0.001**	8.29 (4.26–16.10)	24 (53.3)	**<0.001**	**<0.001**	14.02 (5.34–36.80)	20 (55.5)	**<0.001**	**<0.001**	13.47 (5.06–35.83)
rs1005464 (*BMP2*)													
GG	98 (60.9)	44 (62.0)	Ref.			25 (56.8)	Ref.			22 (62.8)	Ref.		
AG	53 (32.9)	24 (33.8)	>0.999	0.876	0.96 (0.64–1.46)	17 (38.6)	0.587	0.546	1.17 (0.69–2.01)	11 (31.4)	>0.999	0.731	0.88 (0.44–1.77)
AA	10 (6.2)	3 (4.2)	0.755	0.550	0.73 (0.26–2.01)	2 (4.5)	>0.999	0.766	0.82 (0.23–2.92)	2 (5.7)	>0.999	0.790	0.83 (0.22–3.13)
rs17563 (*BMP4*)													
AA	55 (34.6)	23 (32.4)	Ref.			16 (36.4)	Ref.			13 (37.1)	Ref.		
AG	74 (46.5)	29 (40.8)	0.869	0.965	1.01 (0.63–1.60)	18 (40.9)	0.699	0.929	0.97 (0.53–1.76)	12 (34.3)	0.509	0.353	0.70 (0.34–1.46)
GG	30 (18.9)	19 (26.8)	0.333	0.247	1.33 (0.81–2.17)	10 (22.7)	0.817	0.811	1.08 (0.54–2.16)	10 (28.6)	0.476	0.416	1.34 (0.65–2.76)
rs59983488 (*RUNX2*)													
GG	106 (66.7)	51 (73.9)	Ref.			33 (75.0)	Ref.			24 (72.7)	Ref.		
GT	48 (30.2)	14 (20.3)	0.189	0.163	0.68 (0.39–1.16)	7 (15.9)	0.114	0.066	0.46 (0.20–1.05)	8 (24.2)	0.533	0.801	0.90 (0.42–1.92)
TT	5 (3.1)	4 (5.8)	0.480	0.319	1.43 (0.70–2.94)	4 (9.1)	0.228	0.120	1.88 (0.84–4.18)	1 (3)	>0.999	0.836	1.20 (0.20–7.24)
rs1200425 (*RUNX2*)													
GG	51 (32.1)	29 (41.4)	Ref.			17	Ref.			16 (47.0)	Ref.		
AG	81 (50.9)	32 (45.7)	0.272	0.411	0.83 (0.54–1.28)	20	0.452	0.504	0.81 (0.45–1.47)	14 (41.2)	0.154	0.275	0.50 (0.15–1.71)
AA	27 (17.0)	9 (12.9)	0.287	0.336	0.73 (0.39–1.37)	6	0.613	0.569	0.78 (0.34–1.80)	4 (11.8)	0.284	0.130	0.50 (0.20–1.22)
rs3934908 (*SMAD6*)													
CC	45 (28.1)	17 (23.6)	Ref.			8 (18.2)	Ref.			9 (25.0)	Ref.		
CT	85 (53.1)	37 (51.4)	0.734	0.604	1.20 (0.59–2.42)	22 (50.0)	0.519	0.320	1.58 (0.63–3.95)	20 (55.5)	0.829	0.715	1.19 (0.46–3.05)
TT	30 (18.8)	18 (25.0)	0.304	0.153	1.84 (0.78–4.32)	14 (31.8)	0.056	**0.023**	3.25 (1.17–8.99)	7 (19.4)	0.786	0.508	1.47 (0.46–4.70)
rs2119261 (*SMAD6*)													
CC	45 (28.1)	19 (27.1)	Ref.			13 (29.5)	Ref.			9 (26.5)	Ref.		
CT	89 (55.6)	43 (61.4)	0.744	0.476	1.27 (0.65–2.47)	25 (56.8)	>0.999	0.686	1.18 (0.53–2.62)	22 (64.7)	0.677	0.459	1.41 (0.56–3.50)
TT	26 (16.3)	8 (11.4)	0.636	0.366	0.61 (0.21–1.75)	6 (13.6)	0.791	0.694	0.79 (0.24–2.55)	3 (8.8)	0.527	0.397	0.49 (0.09–2.54)

*p^*u*^ was obtained by Fisher’s exact test. p^*m*^ and prevalence ratio (PR) were obtained by Poisson regression adjusted by gender. All comparisons were performed with control group as reference. Bold forms mean *p*-values < 0.05.*

Table 5 shows the diplotype analysis. The diplotype analysis of the studied genetic polymorphisms in *BMP2* demonstrated that individuals carrying TT + AA genotypes (rs235768–rs1005464) had a higher chance to present tooth agenesis (*p* = 0.016; PR = 5.33; 95% CI = 1.36–20.83), third molar agenesis (*p* = 0.013; PR = 10.15; 95% CI = 1.62–63.29), and other agenesis (*p* = 0.020; PR = 7.64; 95% CI = 1.38–42.16) than the control individuals.

**TABLE 5:** Diplotype analysis with *BMP2* gene SNPs by Poisson regression adjusted by gender and ethnicity.

			Control vs. agenesis		Control vs. third molar agenesis			
SNPs	Reference diplotype	Diplotypes	PR (95% CI)	*p*-Value	PR (95% CI)	*p*-Value	PR (95% CI)	*p*-Value
rs235768 and rs1005464	AA + GG	AT + GA	1.04 (0.30–3.60)	0.949	1.68 (0.33–8.55)	0.527	1.17 (0.25–5.38)	0.836
		TT + AA	5.33 (1.36–20.83)	**0.016**	10.15 (1.62–63.29)	**0.013**	7.64 (1.38–42.16)	**0.020**

*PR, prevalence ratio.*

*Bold forms mean *p*-values < 0.05. Other genes were not associated.*

## Discussion

Dental development results from several interactions acting synergistically and antagonistically, leading to tooth epithelium and mesenchyme formation. The process is regulated by different mechanisms involving the expression of several genes (Nieminen, 2009). Mutations and/or genetic polymorphisms in one or more genes involved in the earlier stages of dental development could potentially lead to tooth agenesis. Therefore, in the present study, we replicated genotype–phenotype associations previously observed (Antunes et al., 2012; Gong et al., 2015) and also investigated the association of some novel genes and polymorphisms with isolated tooth agenesis.

In the past decades, innumerous evidence suggests the association between tooth agenesis and oral clefting [revised by Phan et al. (2016)]. Phan et al. (2016) systematically investigated the currently available literature to investigate co-occurrence of tooth agenesis and oral clefts to gain insight into the molecular mechanisms underlying their dual involvement in the development of teeth and facial primordia. The authors concluded that not only the disrupted gene, but even the location of the mutations within the gene can lead to diverse phenotypes, ranging from the isolated form of tooth agenesis to the syndromic one for of oral clefts. In fact, genes involved in oral clefts and in the syndromic form of tooth agenesis are known as a useful approach to select candidate genes for the isolated forms of tooth agenesis (Vieira, 2003). Another important approach to select candidate genes for isolated tooth agenesis is based on the identification of the genes expressed in dental development. So far, more than 300 genes are listed in the database created to catalog genes expressed in different stages of dental development.^5^ Therefore, the genes were selected in our study based on their previous associations and their expression and role in dental development.

Genetic polymorphisms are DNA sequence variations occurring in the genome that are characterized by the existence of at least two variants (Botstein and Risch, 2003). They are involved in many phenotypic differences observed in clinical practice. The selection of the genetic polymorphisms studied here was based on their MAF due to our limited sample size. Previous associations with craniofacial phenotypes were also taken into consideration (as shown in Table 1) and also the function of the genetic polymorphism.

Two genetic polymorphisms (rs17563 and rs235768) selected here are missense variations located within the coding region and produce amino acid changes. The rs17563 in *BMP4* studied here replaced the amino acid valine by alanine at position 152 of the protein. This genetic polymorphism has been widely investigated in oral cleft research. A recent systematic review and comprehensive meta-analysis investigated case-control studies with 2,058 oral cleft cases and 2,557 controls. In their overall analysis, no significant association between the rs17563 polymorphism and the risk of oral cleft was observed, however, their subgroup analysis demonstrated that rs17563 was associated with oral cleft risk in Chinese and Brazilian populations (Bahrami et al., 2020). The polymorphism rs17563 was also associated with isolated tooth agenesis in Brazilians and Chinese (Antunes et al., 2012; Gong et al., 2015). Although we also investigated Brazilians, the lack of association observed in our study may be explained by the fact that these previous studies excluded third molars. Although we also performed a stratified analysis excluding third molars, the sample size could lead to a false-negative result, once it is well-known that BMP4 is important for tooth development and the BMP4 expression pattern coincides with the bud-to-cap stage transition in tooth development (Saadi et al., 2013).

The other missense variant studied here was the rs235768, which is located in BMP2 resulting in an arginine to serine replacement. *BMP2*, another important member of the *BMP* family involved in regulating tooth initiation, can induce human tooth germ cells to differentiate into odontogenic and osteogenic cells (Zhang et al., 2005; Taşlı et al., 2014). In animal models, *BMP2* expressed in the presumptive dental epithelium (Neubüser et al., 1997) could result in the arrest of tooth development after knockdown (Yuan et al., 2015). In fact, our study demonstrated interesting results in both studied genetic polymorphisms in BMP2. To carry the T allele increases the risk to present tooth agenesis. The haplotype and diplotype analysis also showed that rs235768 interacts with the intronic variant rs1005464 and is involved in the risk for any type of tooth agenesis, including third molar agenesis.

Third molars are the most common congenitally missing teeth, followed by premolars and maxillary lateral incisors (Polder et al., 2004). Although third molar agenesis is a phenotype highly prevalent in humans, its etiology has been poorly explored so far. A recent study evaluated a large sample of same sex twins (172 monozygotic and 112 dizygotic) and concluded that a dominant factor for third molar agenesis is genetics (Trakinienë et al., 2018). However, the genes involved in third molar agenesis are still unexplored. Our results suggest that genes/genetic polymorphisms involved in the agenesis of other tooth types could be candidate for third molar agenesis studies.

Mothers against decapentaplegic homolog 6 is known to interact with BMP signaling in the nucleus by interacting with transcription repressors (Wu et al., 2016), including BMP2 (Li et al., 2003). SMAD6 is important for craniofacial development (Estrada et al., 2011; Timberlake et al., 2016). A previous study identified rare *SMAD6* and common *BMP2* alleles involved with craniosynostosis in humans (Timberlake et al., 2016). In our study, genotype and allele distribution of the intronic variant rs3934908 in *SMAD6* was associated with third molar agenesis.

Runt-related transcription factor is well known to be involved in tooth development (Camilleri and McDonald, 2006). RUNX2-deficient mice show an arrest of molar tooth development at the early cap stage, suggesting that RUNX2 is required for the progression of tooth development from the cap stage to the bell stage (D’Souza et al., 1999) and therefore is a candidate for isolated tooth agenesis. In our study, some borderline association was observed for the studied genetic polymorphisms in *RUNX2*, suggesting that future studies should investigate the association of variations in this gene with isolated tooth agenesis in a larger population.

Although our study provides some interesting information regarding the genes involved in the etiology of isolated tooth agenesis, it has some obvious limitations. The limited sample size could lead to a type II error in the analysis of genetic polymorphisms with small effect. Also, the number of selected genetic polymorphisms does not cover the studied genes. Additionally, future studies should also evaluate the role of these genetic polymorphisms in tooth agenesis risk in oral cleft individuals.

In conclusion, our study suggested that genetic polymorphisms in *BMP2* and *SMAD6* are involved in a higher chance to present isolated tooth agenesis.

## Data Availability Statement

The original contributions presented in the study are included in the article/Supplementary Material, further inquiries can be directed to the corresponding authors.

## Ethics Statement

The studies involving human participants were reviewed and approved by Local Ethics Committee (no. 01451418.3.0000.5419). Written informed consent to participate in this study was provided by the participants’ legal guardian/next of kin.

## Author Contributions

EK and CK conceptualize the study. EK, MM, and RS designed and organize the sample recruitment. GM-V and AS performed the sample collection. CR, GM-V, and AS-S performed the laboratory analysis. CR, AS-S, and EP analyzed the data. CR, EK, GM-V, AS-S, and CK wrote the manuscript. EK, PP, and CK funding support. All authors read and approved the final version of the manuscript.

## Conflict of Interest

The authors declare that the research was conducted in the absence of any commercial or financial relationships that could be construed as a potential conflict of interest.

## Publisher’s Note

All claims expressed in this article are solely those of the authors and do not necessarily represent those of their affiliated organizations, or those of the publisher, the editors and the reviewers. Any product that may be evaluated in this article, or claim that may be made by its manufacturer, is not guaranteed or endorsed by the publisher.

## Footnotes

1
http://www.ncbi.nlm.nih.gov/snp/

2
http://snpinfo.niehs.nih.gov/

3
wpcalc.com/en/equilibrium-hardy-weinberg

4
zzz.bwh.harvard.edu/plink

5
http://bite-it.helsinki.fi
