# Supplementary material for: Exploring the Association Between Genetic Polymorphisms in Genes Involved in Craniofacial Development and Isolated Tooth Agenesis
Source: Front Physiol. 2021 Sep 1;12:723105. doi: 10.3389/fphys.2021.723105 (PMC8440976; doi:10.3389/fphys.2021.723105)
Supplement: Supplementary file 1 [file Table_1.DOCX]

| Supplementary Table 1: Characteristics of population | | | | | | | | | | | | |
| --- | --- | --- | --- | --- | --- | --- | --- | --- | --- | --- | --- | --- |
| Variables | | Control Group | | Dental Agenesis | |  | Third Molar Agenesis | |  | Other Agenesis | |  |
|  |  | n | % | n | % | p | n | % | p | n | % | p |
| Total | n = 273 | 187 | 68.5 | 86 | 31.5 | - | 53 | 61.6 | - | 42 | 48.9 | - |
| Gender | Male | 74 | 39.6 | 42 | 48.8 | 0.187 | 27 | 50.9 | 0.157 | 20 | 47.6 | 0.386 |
|  | Female | 113 | 60.4 | 44 | 51.2 |  | 26 | 49.1 |  | 22 | 52.4 |  |
| Ethnicity | Caucasian | 149 | 88.2 | 70 | 84.3 | 0.429 | 42 | 82.4 | 0.343 | 35 | 87.5 | >0.999 |
|  | Black | 20 | 11.8 | 13 | 15.7 |  | 9 | 17.6 |  | 5 | 12.5 |  |
| Center | Curitiba | 110 | 58.8 | 56 | 65.1 | 0.345 | 33 | 62.2 | 0.751 | 27 | 64.3 | 0.602 |
|  | Ribeirão Preto | 77 | 41.2 | 30 | 34.9 |  | 20 | 37.8 |  | 15 | 35.7 |  |
